# Continuous monitoring of trends in pathogen and susceptibility profiles in patients with skin and soft tissue infections (SSTIs)

**DOI:** 10.2478/abm-2023-0043

**Published:** 2023-09-17

**Authors:** Editorial Office of Asian Biomedicine

**Affiliations:** Faculty of Medicine, Chulalongkorn University, Bangkok 10330, Thailand

The skin is colonized by a diverse collection of microorganisms. Most microorganisms coexist with their human hosts without any complications. However, in many conditions, such as impaired host immunity and presence of virulent pathogens, skin and soft tissue infections (SSTIs) can occur [1].

The most common organisms associated with SSTIs are bacteria, but other organisms such as fungi, viruses, *Mycobacterium tuberculosis*, and protozoa may occur [1]. The profile of predominant pathogens may vary based on geographical regions. This is most likely due to evolution of pathogens associated with environmental changes, as well as the volume of antimicrobial use in the areas to control infections in humans and animals. The inappropriate use of antimicrobial agents can cause antimicrobial resistance [2, 3].

The spectrum of clinical manifestations in patients with SSTIs may range from cellulitis, folliculitis, erysipelas, and abscesses, including large furuncles and carbuncles. The clinical spectrum of the SSTIs can range from mild to severe, including toxic shock syndrome, myonecrosis/gas gangrene, and necrotizing fasciitis [4].

Definite diagnosis of pathogens causing SSTIs is necessary for an appropriate choice of antimicrobial regimens. Definite diagnosis will require the isolation of pathogens via cultures of the skin lesions, biopsy with adequate tissues, and blood culture, among others. Isolated pathogens can be identified through specific microbiological analytic techniques such as special stains, molecular techniques, and antigen detection methodologies [1]. Immunocompromised hosts such as patients with immunodeficiencies, HIVs, cancers, and other clinical conditions can pose special diagnostic and therapeutic challenges [1].

Removal of pus and necrotic tissue from the source of infection is needed. The choice of initial treatment depends on the epidemiological trends (community- or hospital-acquired infections), pathogen or pathogens involved, the virulence of pathogens, the seriousness of pathology, the patient's co-morbidities, as well as the symptoms and signs of systemic toxicity. There is an increasing use of rapid diagnostic tests to help in the selection and de-escalation of antimicrobials for SSTIs [4]. Knowledge of local epidemiology and antimicrobial susceptibility patterns of community and hospital strains, as well as the immunological status of the patients, is important. Immunocompromised patients with signs of systemic toxicity may need broad coverage to prevent poor outcomes. In immunocompetent patients with nonpurulent cellulitis without systemic toxicity and risk for methicillin-resistant *Staphylococcus aureus* (MRSA), coverage for low-virulence bacteria such as streptococci and methicillin-sensitive *S. aureus* may be adequate. For most patients with simple skin abscess, oral antibiotic therapy may be sufficient. Immunocompetent patients with purulent cellulitis and signs of possible systemic toxicity or rapid deterioration may require coverage of virulent pathogens such as MRSA [1].

Severe SSTIs usually require intensive care, effective source control, and presumptive broad-spectrum antibiotics initially. Rapid diagnostic tests may be essential to help select and de-escalate initial antibacterial regimens [3].

Clinical prediction scores may help to predict patients who do not require antimicrobials directed against virulent pathogens such as MRSA [5]. The immune status of some patients has been shown to be important in the clinical outcomes of some, but not all, types of SSTIs. The debate regarding the benefits of intravenous immunoglobulin continues to be waged in recent literatures [6].

The duration of antimicrobial therapy depends on the severity of infections and their response to treatment. Symptomatic improvement usually occurs within 24–48 h, although visible improvement of skin manifestations can take 72 h or longer. Worsening systemic symptoms should prompt a search for possible reasons for treatment failure. Considerations include the presence of deeper infections, inadequate antibiotic penetration, or incorrect diagnosis [7].

Khan et al. [8] in this volume report the profile of bacterial isolates of superficial and deep-seated SSTIs and their antimicrobial susceptibility. They noticed that the predominant sources of SSTIs are gram-negative rods with very high resistance against oral antibiotics. Moreover, multidrug-resistant *Escherichia. coli* and *Klebsiella* spp., MRSA, and organisms with resistance to carbapenem have emerged. Knowledge of these organisms is important to guide treatment of SSTIs.

In conclusion, SSTIs are common, and their management can be complex due to regional variation in predominant pathogens and antimicrobial resistance patterns, as well as variations in host immune responses [3]. Continuous monitoring of trends in pathogen and susceptibility profiles is important to advise local public health efforts regarding the appropriate treatment of SSTIs [3, 9]. The need for a One Health approach to restrict the emergence of antimicrobial-resistant virulent pathogens cannot be overemphasized [9].
